# Analysis of BMP4 and BMP7 signaling in breast cancer cells unveils time-dependent transcription patterns and highlights a common synexpression group of genes

**DOI:** 10.1186/1755-8794-4-80

**Published:** 2011-11-25

**Authors:** Alejandra Rodriguez-Martinez, Emma-Leena Alarmo, Lilli Saarinen, Johanna Ketolainen, Kari Nousiainen, Sampsa Hautaniemi, Anne Kallioniemi

**Affiliations:** 1 Laboratory of Cancer Genetics, Institute of Biomedical Technology, University of Tampere and Centre for Laboratory Medicine, Tampere University Hospital, Finland; 2 Computational Systems Biology Laboratory, Genome-Scale Biology Research Program, Institute of Biomedicine, University of Helsinki, Finland

**Keywords:** bone morphogenetic protein, breast cancer, BMP4, BMP7, expression microarray

## Abstract

**Background:**

Bone morphogenetic proteins (BMPs) are members of the TGF-beta superfamily of growth factors. They are known for their roles in regulation of osteogenesis and developmental processes and, in recent years, evidence has accumulated of their crucial functions in tumor biology. BMP4 and BMP7, in particular, have been implicated in breast cancer. However, little is known about BMP target genes in the context of tumor. We explored the effects of BMP4 and BMP7 treatment on global gene transcription in seven breast cancer cell lines during a 6-point time series, using a whole-genome oligo microarray. Data analysis included hierarchical clustering of differentially expressed genes, gene ontology enrichment analyses and model based clustering of temporal data.

**Results:**

Both ligands had a strong effect on gene expression, although the response to BMP4 treatment was more pronounced. The cellular functions most strongly affected by BMP signaling were regulation of transcription and development. The observed transcriptional response, as well as its functional outcome, followed a temporal sequence, with regulation of gene expression and signal transduction leading to changes in metabolism and cell proliferation. Hierarchical clustering revealed distinct differences in the response of individual cell lines to BMPs, but also highlighted a synexpression group of genes for both ligands. Interestingly, the majority of the genes within these synexpression groups were shared by the two ligands, probably representing the core molecular responses common to BMP4 and BMP7 signaling pathways.

**Conclusions:**

All in all, we show that BMP signaling has a remarkable effect on gene transcription in breast cancer cells and that the functions affected follow a logical temporal pattern. Our results also uncover components of the common cellular transcriptional response to BMP4 and BMP7. Most importantly, this study provides a list of potential novel BMP target genes relevant in breast cancer.

## Background

Bone morphogenetic proteins (BMPs) are extracellular ligand molecules that belong to the transforming growth factor β (TGF-β) superfamily. To date, 21 members of the human BMP family have been identified [1]. BMPs regulate transcription of target genes by signaling through type I and II transmembrane serine-threonine receptors. Binding of the ligand to the type II receptor elicits phosphorylation of the type I receptor, which, as a result, is able to phosphorylate other molecules and transmit the signal. In the canonical BMP pathway, the type I receptor phosphorylates receptor-regulated SMAD (homologue of Drosophila Mothers Against Decapentaplegic) proteins (R-SMADs, SMAD-1/5/8), which then bind to the common mediator SMAD4; the resulting SMAD complex translocates to the nucleus to regulate transcription of target genes [1]. The signals generated by BMPs in the cell membrane may be also transferred into the cell via ERK, JNK and p38 mitogen-activated protein kinases (MAPK) [2,3]. Moreover, there is crosstalk between BMP signaling and other cellular signaling cascades, such as the Wnt, JAK/STAT and Notch pathways [4-6].

BMPs were first identified as inducers of ectopic bone formation *in vivo *[7] but were later found to be crucial multifunctional regulators of development [8]. During the last decade, the role of BMPs in cancer development has gained increasing interest [9-11]. The importance of BMP4 and BMP7 in breast cancer was highlighted in a survey of seven BMPs: these two ligands had the highest expression levels and were the most frequently expressed among 22 cell lines and 39 primary tumor samples [12]. The expression of BMP4 and BMP7 in breast cancer also has been demonstrated in several other reports [13-17]. Interestingly, BMP7 protein expression in primary breast tumors has been associated with accelerated bone metastasis formation and served as an independent prognostic factor for early bone metastasis in a study based on a set of 409 patient samples [15] though, with a smaller set of 67 patient samples, this association was not established [18].

The functional significance of BMP4 and BMP7 in breast cancer has been studied predominantly through the use of *in vitro *models. BMP4 was shown to inhibit cell proliferation in a panel of breast cancer cell lines by inducing a G1 cell cycle arrest [14]. The effects of exogenous BMP4 on breast cancer cell migration and invasion have also been studied. For the most part, the data suggest promotion of these cellular abilities by BMP4 in several breast cancer cell lines and in normal breast epithelial cells [14,19], while a study in which only MDA-MB-231 cells were analyzed reported the opposite phenotype [20]. For BMP7, the results from different reports and different cell lines are more variable. *In vitro *examination of BMP7 manipulation have revealed cell line-specific effects on cell proliferation, migration and invasion; BMP7 induces all of these parameters in MDA-MB-231 cells and inhibits cellular proliferation in several other cell lines [21]. In opposition, in an *in vivo *xenograft mouse model of MDA-MB-231 cells, BMP7 reduced tumor growth as well as the formation and growth of bone metastases [18].

In spite of the many years since the discovery of BMPs and being currently a very active topic in cancer research, little is known about their target genes in tumor conditions. The present study was designed to gain knowledge in this topic, by exploring the effects of BMP4 and BMP7 signaling on gene transcription in seven breast cancer cell lines and throughout a 6-point time series, using a genome-wide approach. We characterized the transcriptional response of breast cancer cells to BMP signaling in an analysis that included a temporal dimension and the comparison of different cell lines and two BMP ligands. Finally and most importantly, we report novel potential BMP target genes relevant in breast cancer.

## Methods

### Breast cancer cell lines

Seven breast cancer cell lines (HCC1954, MDA-MB-361, ZR-75-30, HCC1419, SK-BR-3, MDA-MB-231 and T-47D) were purchased from the American Type Culture Collection (ATCC, Manassas, VA) and cultured according to the recommended conditions except for MDA-MB-231 and T-47D, for which the concentration of FBS in culture media were 1% and 5%, respectively.

### BMP4 and BMP7 treatments

**Figure 1:**
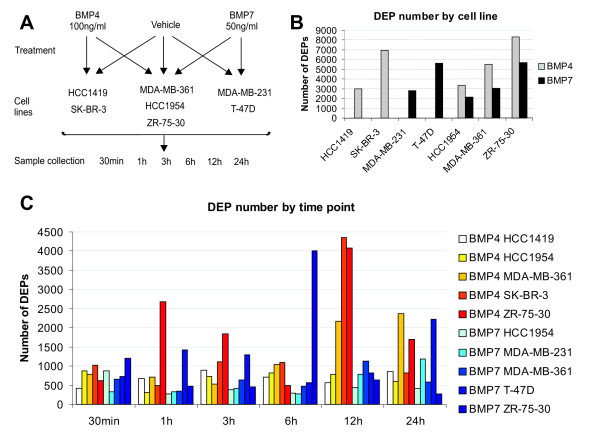
**Experimental workflow and numbers of differentially expressed probes (DEPs) resulting from BMP4 or BMP7 treatment**. (A) Seven breast cancer cell lines were cultured on 24-well plates, allowed to adhere for 24 h, and treated with the BMP ligand or vehicle for 30 min, 1 h, 3 h, 6 h, 12 h, and 24 h. Experiments were performed in triplicate, and collected cells were pooled. (B) The expression data from each cell line were individually filtered according to the following criteria: differential expression of at least 2-fold at a minimum of one time point. The number of DEPs per cell line is represented. (C) The expression data from individual time points of every cell line were filtered according to a 2-fold cutoff in expression change. The number of DEPs per time point is shown.

Recombinant human BMP4 and BMP7 proteins were purchased from R&D Systems (Minneapolis, MN). Three cell lines (HCC1954, MDA-MB-361 and ZR-75-30) were treated with both BMP4 (100 ng/ml) and BMP7 (50 ng/ml) separately. HCC1419 and SK-BR-3 cell lines received only BMP4 treatment (100 ng/ml), while MDA-MB-231 and T-47D were treated only with BMP7 (50 ng/ml). Cells were seeded on 24-well plates, allowed to adhere for 24 h, and treated with the BMP ligand or vehicle for 30 min, 1 h, 3 h, 6 h, 12 h and 24 h (Figure 1A). Experiments were performed in triplicate and collected cells were pooled.

### Microarrays

Total RNA was extracted using the RNeasy Mini Kit (Qiagen, Valencia, CA) and the quality of RNA was validated using the Agilent RNA 6000 Nano Kit (Agilent Technologies, Palo Alto, CA, USA). Total RNA (500 ng) was used to generate fluorescent Cy-3-(vehicle treated cells) or Cy-5-labeled cRNA (BMP4- or BMP7-treated cells) using the Agilent Low RNA Input Fluorescence Linear Amplification Kit (Agilent Technologies). The labeled cRNAs were hybridized to the 44 K Whole Human Genome oligo microarrays (Agilent Technologies) according to the manufacturer's protocol. Microarray slides were scanned (Agilent Microarray Scanner) after hybridization, and data were extracted using the Feature Extraction software, version A.7.5.1 (Agilent Technologies). The microarray data has been submitted to the GEO database (accession number GSE31605).

### Data analysis

The microarray data were first subjected to linear normalization to allow comparison between arrays. All probes were compared to the reference sequence using BLAST (v.2.2.23). Ensembl IDs for the probes were obtained by examining the probe's genomic location. The Ensembl *Homo sapiens *database version 60.37e was used. This process resulted in the annotation of 84% of all the probes. A total of 66% of the probes mapped uniquely to genes and 16% mapped to multiple genes.

In order to determine differentially-expressed genes, expression data were subjected to three types of filtering: cell line-specific, time point-specific and general filtering. Cell line-specific filtering was done separately for each cell line, following the criteria of a differential expression of at least 2-fold in a minimum of one time point. In the time point-specific filtering, data from each time point were independently filtered according to a 2-fold expression change cutoff. General filtering was performed on all the data from all the cell lines together (separately for BMP4 and BMP7) following the next criteria: probes with a differential expression of at least 3-fold in at least three events and/or 2-fold in at least four events were considered for subsequent analysis. An event refers to any time point of any cell line, resulting in a maximum number of 30 events (5 cell lines and 6 time points per cell line). The data sets produced by general filtering were further hand-annotated to reduce the number of probes with multiple annotations. Uniquely annotated probes are designated hereafter as "genes, " whereas the terms "probe" and "genetic element" refer to multiple annotated probes or any data including them. The gene lists resulting from general filtering were ranked according to the number of events in which they showed regulation. Furthermore, all the probes derived from general filtering were subjected to hierarchical clustering using correlation metrics, agglomerative strategy and average linkage method.

Enrichments of gene ontology (GO) terms were performed on several data sets applying Fisher's exact test and using all genes present on the microarray as a reference [22]. In all the GO enrichment analyses, only probes with unique annotation were used.

A model-based clustering method [23] that allows the finding of clusters of genes with similar expression profiles was performed using MCLUST R package [24]. This method was applied to data resulting from cell line-specific filtering. Data were log2-transformed and scaled to unit length. In the model-based clustering method, the clusters are considered to be groups displaying multivariate distributions. Several models were fitted to the data. The selections of the best model and the number of clusters were made based upon maximizing Bayesian Information Criterion (BIC) values for the specific model and number of clusters that best represented the data. The data analyses were performed using the Anduril data analysis framework [25] and R [26].

## Results

The aim of this study was to uncover the transcriptional responses of BMP4 and BMP7 signaling in breast cancer. To this end, we selected breast cancer cell lines with low endogenous expression of BMP4 (HCC1419, SK-BR-3), BMP7 (MDA-MB-231, T-47D) or both (HCC1954, MDA-MB-361, ZR-75-30) [12,14] and treated them with the corresponding BMP ligand (rhBMP4 or rhBMP7) and vehicle controls (Figure 1A). Global gene expression levels were analyzed at six different time points from 30 min to 24 h in order to reveal the temporal patterns of transcriptional changes.

### Overall transcriptional response to BMP4 and BMP7 treatment

Due to the multidimensional nature of our data, we used three different filtering approaches, each of them allowing analysis from a different perspective. Cell line-specific analysis of the expression data evidenced considerable variation in the number of differentially expressed probes (DEPs) from one cell line to another (Figure 1B), implicating distinct differences in their transcriptional response to BMPs. Further evaluation of these results revealed that BMP4 treatment resulted in greater amounts of DEPs than BMP7 (average number of DEPs per cell line: 5, 469 versus 3, 898 for BMP4 and BMP7, respectively; Figure 1B). This finding could not be explained by the differences in cell lines used to study the two ligands, as a similar outcome was observed in the three lines treated with both BMP4 and BMP7 (HCC-1954, MDA-MB-361 and ZR-75-30). Time point-specific filtering revealed clear temporal variation in the number of DEPs (Figure 1 C). Generally, there was a tendency towards a greater amount of DEPs at later time points. In order to focus our attention especially on those genes whose expression was most consistently and extensively affected by BMP4 and BMP7 signaling, we performed a general filtering of the expression data according to the following criteria: fold change (FC) ≥ +/-3 in at least 3 events and/or FC ≥ +/-2 in at least 4 events. This resulted in the identification of 2, 421 and 1, 263 differentially expressed gene elements (1, 678 and 905 uniquely annotated probes) for BMP4 and BMP7 experiments respectively, further evidencing a more prominent effect of BMP4 than BMP7 on gene transcription.

**Figure 2:**
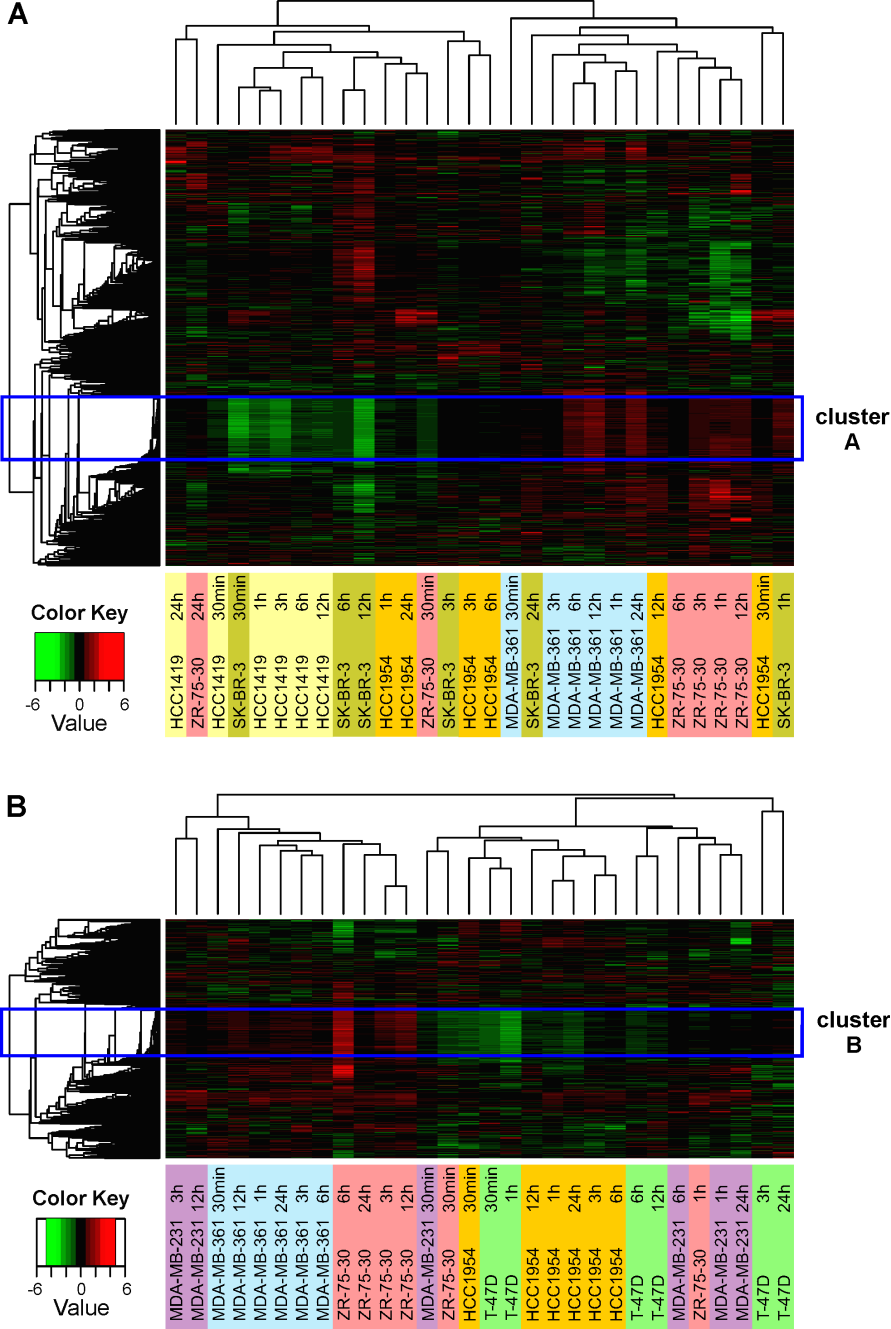
**Unsupervised hierarchical clustering of microarray data**. The data sets resulting from general filtering (2, 421 probes for (A) BMP4 and 1, 263 probes for (B) BMP7) were subjected to this analysis. For both ligands, there is an evident cluster of gene elements showing very highly correlated expression patterns throughout the samples (gene clusters A and B, blue boxes).

Unsupervised hierarchical clustering on the data sets resulting from general filtering (BMP4 and BMP7 separately) revealed that the samples originating from a particular cell line mainly clustered together (Figure 2), suggesting considerable variation in the response of individual cell lines to BMPs. The most obvious examples are MDA-MB-361, ZR-75-30, and HCC1419 (for BMP4) as well as MDA-MB-361 and HCC1954 (for BMP7). Clustering according to time point was an uncommon phenomenon, but it was observed for the samples derived from MDA-MB-231, ZR-75-30, HCC1954 and T-47D after 30 min of BMP7 treatment (Figure 2B). At the probe level, both BMP4 and BMP7 hierarchical trees revealed a small subset of gene elements that clustered tightly together (named clusters A and B hereafter, Figure 2, blue boxes). However, it is important to note that the expression of the genes in these clusters was not altered in a similar fashion in all the cell lines; rather, they were upregulated in some cell lines and downregulated in others, showing diverse temporal patterns. For both ligands, these clusters appeared to dictate the division of the samples into two major tree branches (Figure 2).

In order to obtain a general view of the cellular functions regulated as a result of BMP4 and BMP7 signaling in breast cancer cell lines, we performed a GO enrichment analysis on the data sets resulting from general filtering. As might have been expected, functional categories related to regulation of transcription were among the most highly enriched for both ligands. Additionally, genes involved in organ development were abundantly regulated as a result of stimulation with either ligand. In this regard, BMP4 seemed more often to regulate genes involved in skeletal system development (Table 1), while BMP7, on the other hand, appeared to regulate genes involved in epithelial development, neurogenesis and tube development (Table 2).

**Table 1:** Enriched gene ontology categories for differentially expressed genes as a result of BMP4 treatment.

Category	Number of genes	*p*-Value
*Biological process*		
GO:0006355: regulation of transcription, DNA-dependent	240	0.005
GO:0009888: tissue development	128	0.014
GO:0001501: skeletal system development	53	0.031
GO:0030154: cell differentiation	237	0.041
		
*Molecular function*		
GO:0043565: sequence-specific DNA binding	94	0.043
GO:0003700: sequence-specific DNA binding transcription factor activity	133	0.046

**Table 2:** Enriched gene ontology categories for differentially expressed genes as a result of BMP7 treatment.

Category	Number of genes	*p*-Value
*Biological process*		
GO:0030182: neuron differentiation	55	0.024
GO:0048730: epidermis morphogenesis	8	0.025
GO:0051239: regulation of multicellular organismal process	88	0.025
GO:0045944: positive regulation of transcription from RNA polymerase II promoter	42	0.028
GO:0030855: epithelial cell differentiation	24	0.031
GO:0016481: negative regulation of transcription	50	0.033
GO:0035295: tube development	32	0.042
		
*Molecular function*		
GO:0043565: sequence-specific DNA binding	57	0.024
GO:0003705: RNA polymerase II transcription factor activity, enhancer binding	10	0.025
GO:0016564: transcription repressor activity	38	0.038

### A common synexpression group of genes regulated in response to BMP4 and BMP7 signaling

**Figure 3:**
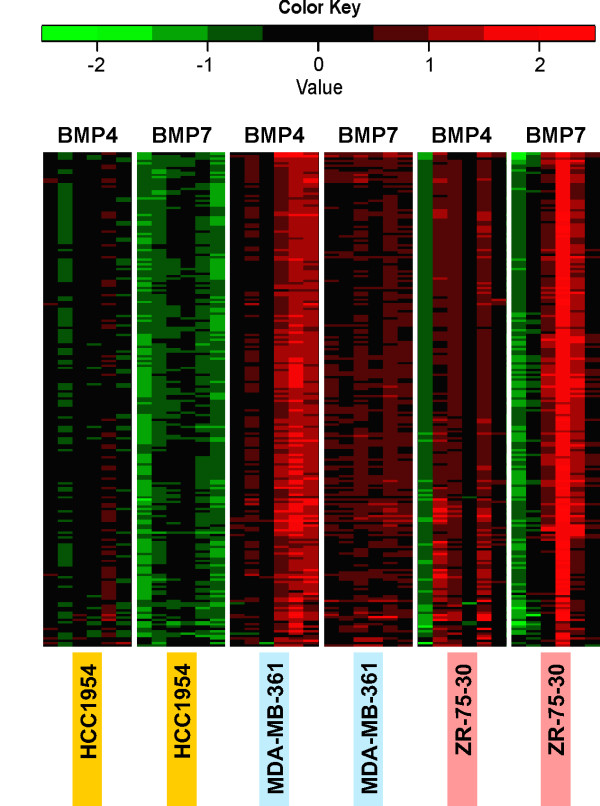
**Hierarchical clustering heat map of group C genes with supervised clustering at the sample level**. Data from each cell line are grouped, and time points are arranged in temporal order from left to right: 30 min, 1 h, 3 h, 6 h, 12 h and 24 h.

As mentioned above, hierarchical clustering unveiled gene clusters A (BMP4, containing 329 probes) and B (BMP7, 228 probes) with highly correlated expression patterns (Figure 2, blue boxes). Interestingly, of all the probes contained in clusters A and B, 210 (154 genes with known and unique annotation, named group C hereafter, Additional file 1) were present in both clusters and thus represent shared BMP target genes. Direct comparisons of the expression patterns of group C probes in the three cell lines (HCC1954, MDA-MB-361 and ZR-75-30) treated with both ligands revealed elements of similarity between BMP4 and BMP7 response in the same cell line but high variability between different cell lines (Figure 3). For example, these genes were commonly upregulated in MDA-MB-361 while downregulated in HCC1954. The GO enrichment analysis unveiled 23 enriched biological process terms, of which 21 could be classified into two functional categories, namely development and morphogenesis (16 terms) and gene expression (5 terms) (Additional file 2).

### Temporal patterns of transcriptional response to BMP signaling

**Figure 4:**
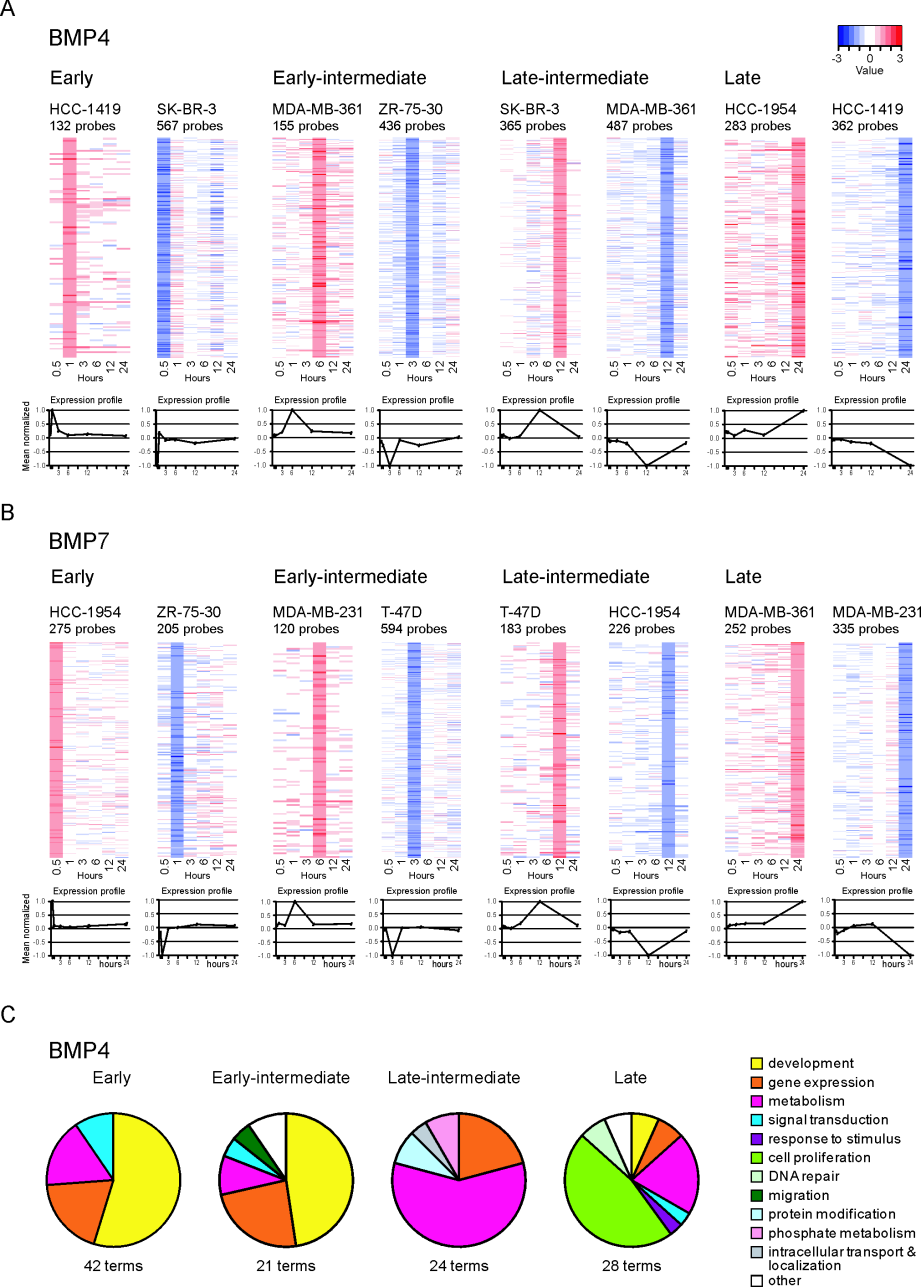
**Time series analyses**. Representative clusters from the four temporal categories are shown for BMP4 (A) and BMP7 (B). For each cluster, the upper figure shows the levels of differential expression through the time series for every probe. The lower chart represents the average value of differential expression for all the probes in the cluster along the time scale. The number of probes in each cluster is indicated under the cell line name. (C) GO analyses of the four temporal categories were performed, and data from the five cell lines were combined. The enriched GO terms for BMP4 are depicted.

Model-based clustering analysis of the expression data was performed to distinguish clusters of genes with similar temporal profiles of expression. With this method we identified 12 to 22 probe clusters for each cell line and these clusters could be subsequently classified into four main categories (Tables 3 and 4 and Additional file 3). Gene elements that were first regulated at 30 min or 1 h were classified as early, 3 h or 6 h early-intermediate, and 12 h late-intermediate responders, regardless of their expression at later time points. Late responders included those probes differentially expressed exclusively at the 24 h time point. Representative examples of clusters in the different temporal categories are depicted in Figure 4 A and 4B.

**Table 3:** Summary of the temporal clusters obtained from the analysis of BMP4 data.

	Early		Early-intermediate		Late-intermediate		Late		Undetermined	
	
	cluster ID	genes/cluster	cluster ID	genes/cluster	cluster ID	genes/cluster	cluster ID	genes/cluster	cluster ID	genes/cluster
HCC1419	2	256	1	273	8	189	6	362	3	178
(14 clusters)	5	148	4	341			11	353	7	152
	10	107	14	31					9	224
	12	157								
	13	132								
*Total*		*800*		*654*		*189*		*715*		*554*

HCC1954	2	265	4	256	1	158	7	283		
(12 clusters)	5	353	9	258	3	419	10	156		
	6	108	11	244						
	8	110	12	251						
*Total*		*836*		*1009*		*577*		*439*		

MDA-MB-361	6	277	3	155	8	322	1	557	5	289
(14 clusters)	13	215	7	177	9	487	2	483	10	362
	14	355	12	270	11	554	4	564		
*Total*		*847*		*602*		*1363*		*1604*		*651*

SK-BR-3	1	77	2	232	3	235	4	252		
(17 clusters)	6	70	10	402	7	365	5	300		
	11	148	14	383	8	1322				
	13	567	16	332	9	1104				
	17	51			12	95				
					15	508				
*Total*		*913*		*1349*		*3629*		*552*		

ZR-75-30	1	601	9	140	4	2201	8	369	2	10
(18 clusters)	5	107	10	780	14	185	11	688	3	10
	12	53	18	436	16	809			6	9
	15	227							7	9
	17	1363							13	17
*Total*		*2351*		*1356*		*3195*		*1057*		*55*

Gene clusters were classified in four temporal categories (early, early-intermediate, late-intermediate and late). Clusters with unclear profiles were classified as undetermined. The cluster ID column contains the ID number of the cluster.

**Table 4:** Summary of the temporal clusters obtained from the analysis of BMP7 data.

	Early		Early-intermediate		Late-intermediate		Late		Undetermined	
	
	cluster ID	genes/cluster	cluster ID	genes/cluster	cluster ID	genes/cluster	cluster ID	genes/cluster	cluster ID	genes/cluster
HCC1954	2	32	4	66	3	226	1	197	6	119
(14 clusters)	5	324	12	113	10	116			13	113
	7	113	14	149						
	8	35								
	9	275								
	11	111								
*Total*		*890*		*328*		*342*		*197*		*232*

MDA-MB-231	6	97	4	41	2	175	1	335		
(16 clusters)	7	27	9	120	5	321	3	512		
	10	108	11	101	16	144	8	140		
	12	125	14	147						
	13	52	15	85						
*Total*		*409*		*494*		*640*		*987*		

MDA-MB-361	7	240	2	177	1	379	6	252		
(12 clusters)	8	193	3	235	5	328				
	11	258	4	230	9	58				
			10	336	12	73				
*Total*		*691*		*978*		*838*		*252*		

T-47D	2	767	8	212	1	183	5	1265	14	7
(14 clusters)	3	127	9	594	10	377	6	559		
	4	38	12	476						
	7	414								
	11	163								
	13	88								
*Total*		*1597*		*1282*		*560*		*1824*		*7*

ZR-75-30	3	205	2	169	11	105	14	87	1	8
(22 clusters)	5	286	4	30	12	196			7	16
	13	114	6	1544					9	37
	15	216	8	35					17	23
	20	250	10	1493					21	14
	22	36	16	147						
			18	85						
			19	336						
*Total*		*1107*		*3839*		*301*		*87*		*98*

Gene clusters were classified in four temporal categories (early, early-intermediate, late-intermediate and late). Clusters with unclear profiles were classified as undetermined. The cluster ID column contains the ID number of the cluster.

We had already explored the biological functions of the genes differentially expressed upon BMP treatment. As we were now able to temporally classify the genes, we became interested in evaluating whether temporal patterns of expression and gene function could be related. Therefore, we grouped the clusters from each temporal category (early, early-intermediate, late-intermediate, and late) and performed GO enrichment analyses on the genes within these groups (Additional file 3). Finally, results from the different cell lines were combined. Although enriched GO terms were not found in every temporal stage of every cell line, many GO terms were enriched in the four temporal categories for BMP4 (Figure 4 C). Of all the functional terms enriched throughout the experiments, those related to development were especially abundant in the early and early-intermediate phases. Terms connected with regulation of gene expression appeared at all times, although they were less abundant at the late stage of 24 h. Metabolism-associated terms were also present throughout the experiment, but were most prominent at late-intermediate time points. Signal transduction appeared to be an affected biological process in all but the late-intermediate phases; it seemed most profoundly altered during early stages. Many terms related to cell proliferation and DNA repair were enriched among late responder genes, but it should be noted that all these terms emerged from one single cell line, MDA-MB-361. The results from BMP7 data were very limited and may be summarized as an enrichment of genes involved in regulation of metabolism and gene expression among the early-intermediate and late time categories.

### Potential novel BMP target genes in breast cancer

One of the main goals of this study was to identify new BMP4 and BMP7 target genes relevant in breast cancer. In order to distinguish those genes most often and ubiquitously regulated by BMP treatments, differentially expressed genes resulting from general filtering (1, 678 and 905 for BMP4 and BMP7 experiments, respectively) were ranked according to the number of times a gene was up- and downregulated throughout the series of cell lines and time points (Tables 5 and 6 and Additional file 4). It is interesting that although the proportion of up- and down-regulation events was roughly equal when considering all the differentially expressed genes, induction of gene transcription clearly prevailed over inhibition when considering the 100 top-ranked genes (75% and 73% of the events for BMP4 and BMP7, respectively). For BMP4, 80 genes were regulated in 10 or more events, while for BMP7, the analogous number of genes was 29 (Tables 5 and 6). Out of the 30 possible regulation events for a single gene (5 cell lines and 6 time points), the actual maximums were 23 events (*PTPRG*; protein tyrosine phosphatase, receptor type, G) for BMP4 and 19 events (*GNRHR*; gonadotropin-releasing hormone receptor) for BMP7 (Tables 5 and 6); all of these were upregulation events. As expected, several members of the Id family of inhibitors of DNA binding, well-known targets of BMPs [27], were strongly induced by both BMP ligands. In addition to *PTPRG *and *GNRHR*, other genes strongly upregulated by both ligands included *APOC2 *(apolipoprotein C-II) and an as-yet unnamed gene encoding an uncharacterized protein (*C12orf42*). *DUSP2 *(dual specificity phosphatase 2) and *MAP3K5 *(mitogen-activated protein kinase kinase kinase 5) were highly induced only by BMP4. BMP7, on the other hand, strongly promoted the expression of genes including *PBX1 *(pre-B-cell leukemia homeobox 1) and *ZSCAN4 *(zinc finger and SCAN domain containing 4), which were not among the genes most intensely regulated by BMP4.

**Table 5:** Top most regulated genes after BMP4 signaling.

			# regulated events		
Gene Id	Gene name	Gene description	Up	Down	Total
ENSG00000144724	PTPRG	protein tyrosine phosphatase, receptor type, G	23	0	23
ENSG00000125968	ID1	inhibitor of DNA binding 1, dominant negative helix-loop-helix protein	19	0	19
ENSG00000179088	C12orf42	uncharacterized protein C12orf42	19	0	19
ENSG00000158050	DUSP2	dual specificity phosphatase 2	18	0	18
ENSG00000109163	GNRHR	gonadotropin-releasing hormone receptor	18	0	18
ENSG00000234906	APOC2	apolipoprotein C-II	18	0	18
ENSG00000117318	ID3	inhibitor of DNA binding 3, dominant negative helix-loop-helix protein	17	0	17
ENSG00000115738	ID2	inhibitor of DNA binding 2, dominant negative helix-loop-helix protein	16	0	16
ENSG00000172201	ID4	inhibitor of DNA binding 4, dominant negative helix-loop-helix protein	16	0	16
ENSG00000197442	MAP3K5	mitogen-activated protein kinase kinase kinase 5	15	0	15
ENSG00000127129	EDN2	endothelin 2	15	0	15
ENSG00000164850	GPER	G protein-coupled estrogen receptor 1	15	0	15
ENSG00000181638	ZFP41	zinc finger protein 41 homolog (mouse)	15	0	15
ENSG00000181626	ANKRD62	ankyrin repeat domain 62	15	0	15
ENSG00000187957	DNER	delta/notch-like EGF repeat containing	14	0	14
ENSG00000238243	OR2W3	olfactory receptor, family 2, subfamily W, member 3	14	0	14
ENSG00000164683	HEY1	hairy/enhancer-of-split related with YRPW motif 1	14	0	14
ENSG00000157322	CLEC18A	C-type lectin domain family 18, member A	13	1	14
ENSG00000247097	C14orf184	Putative uncharacterized protein C14orf184	8	6	14
ENSG00000212124	TAS2R19	taste receptor, type 2, member 19	9	4	13
ENSG00000186115	CYP4F2	cytochrome P450, family 4, subfamily F, polypeptide 2	6	7	13
ENSG00000176472	ZNF575	zinc finger protein 575	7	6	13
ENSG00000181722	ZBTB20	zinc finger and BTB domain containing 20	7	6	13
ENSG00000163827	LRRC2	leucine rich repeat containing 2	12	0	12
ENSG00000214049	UCA1	urothelial cancer associated 1	12	0	12
ENSG00000132854	KANK4	KN motif and ankyrin repeat domains 4	12	0	12
ENSG00000163749	CCDC158	coiled-coil domain containing 158	12	0	12
ENSG00000100029	PES1	pescadillo homolog 1, containing BRCT domain (zebrafish)	0	12	12
ENSG00000120645	IQSEC3	IQ motif and Sec7 domain 3	0	12	12
ENSG00000122852	SFTPA1	surfactant protein A1	9	3	12
ENSG00000052802	SC4MOL	sterol-C4-methyl oxidase-like	9	3	12
ENSG00000197532	OR6Y1	olfactory receptor, family 6, subfamily Y, member 1	8	4	12
ENSG00000078328	RBFOX1	RNA binding protein, fox-1 homolog (C. elegans) 1	8	4	12
ENSG00000185010	F8	coagulation factor VIII, procoagulant component	8	4	12
ENSG00000115756	HPCAL1	hippocalcin-like 1	8	4	12
ENSG00000122859	NEUROG3	neurogenin 3	5	7	12
ENSG00000186810	CXCR3	chemokine (C-X-C motif) receptor 3	6	6	12
ENSG00000168874	ATOH4	atonal homolog 8 (Drosophila)	11	0	11
ENSG00000040731	CDH10	cadherin 10, type 2 (T2-cadherin)	11	0	11
ENSG00000168930	TRIM49	tripartite motif-containing 49	11	0	11
ENSG00000104863	LIN7B	lin-7 homolog B (C. elegans)	11	0	11
ENSG00000113391	FAM172A	microRNA 2277	11	0	11
ENSG00000162614	NEXN	nexilin (F actin binding protein)	11	0	11
ENSG00000120693	SMAD9	SMAD family member 9	11	0	11
ENSG00000176907	C8orf4	Uncharacterized protein C8orf4 (Thyroid cancer protein 1)(TC-1)	10	1	11
ENSG00000085224	ATRX	alpha thalassemia/mental retardation syndrome X-linked	10	1	11
ENSG00000163743	RCHY1	ring finger and CHY zinc finger domain containing 1	9	2	11
ENSG00000006007	GDE1	glycerophosphodiester phosphodiesterase 1	9	2	11
ENSG00000114850	SSR3	signal sequence receptor, gamma (translocon-associated protein gamma)	9	2	11
ENSG00000109472	CPE	carboxypeptidase E	9	2	11
ENSG00000106608	URGCP	upregulator of cell proliferation	2	9	11
ENSG00000064201	TSPAN32	tetraspanin 32	8	3	11
ENSG00000212128	TAS2R13	taste receptor, type 2, member 13	8	3	11
ENSG00000092871	RFFL	ring finger and FYVE-like domain containing 1	7	4	11
ENSG00000139880	CDH24	cadherin 24, type 2	4	7	11
ENSG00000039319	ZFYVE16	zinc finger, FYVE domain containing 16	4	7	11
ENSG00000206052	DOK6	docking protein 6	6	5	11
ENSG00000050165	DKK3	dickkopf homolog 3 (Xenopus laevis)	5	6	11
ENSG00000115844	DLX2	distal-less homeobox 2	10	0	10
ENSG00000178343	SHISA3	shisa homolog 3 (Xenopus laevis)	10	0	10
ENSG00000123329	ARHGAP9	Rho GTPase activating protein 9	10	0	10
ENSG00000145287	PLAC8	placenta-specific 8	10	0	10
ENSG00000187634	SAMD11	sterile alpha motif domain containing 11	10	0	10
ENSG00000167962	ZNF598	zinc finger protein 598	10	0	10
ENSG00000003509	C2orf56	Protein midA homolog, mitochondrial Precursor	8	2	10
ENSG00000143153	ATP1B1	ATPase, Na+/K+ transporting, beta 1 polypeptide	8	2	10
ENSG00000189079	ARID2	AT rich interactive domain 2 (ARID, RFX-like)	2	8	10
ENSG00000162706	CADM3	cell adhesion molecule 3	7	3	10
ENSG00000147488	ST18	suppression of tumorigenicity 18 (breast carcinoma) (zinc finger protein)	7	3	10
ENSG00000182175	RGMA	RGM domain family, member A	7	3	10
ENSG00000163623	NKX6-1	NK6 homeobox 1	3	7	10
ENSG00000144559	C3orf31	MMP37-like protein, mitochondrial Precursor	6	4	10
ENSG00000153002	CPB1	carboxypeptidase B1 (tissue)	6	4	10
ENSG00000181965	NEUROG1	neurogenin 1	6	4	10
ENSG00000171564	FGB	fibrinogen beta chain	6	4	10
ENSG00000132612	VPS4A	vacuolar protein sorting 4 homolog A (S. cerevisiae)	6	4	10
ENSG00000183023	SLC8A1	solute carrier family 8 (sodium/calcium exchanger), member 1	4	6	10
ENSG00000166748	AGBL1	ATP/GTP binding protein-like 1	5	5	10
ENSG00000204882	GPR20	G protein-coupled receptor 20	5	5	10
ENSG00000159216	RUNX1	runt-related transcription factor 1	5	5	10

Differentially expressed genes resulting from the general filtering of BMP4 data (1, 678) were ranked according to the number of times a gene was up- and downregulated throughout the series of cell lines and time points. Only those genes with total rank value of at least 10 are listed.

**Table 6:** Top most regulated genes after BMP7 signaling.

			# regulated events		
Gene Id	Gene name	Gene description	Up	Down	Total
ENSG00000109163	GNRHR	gonadotropin-releasing hormone receptor	19	0	19
ENSG00000157322	CLEC18A	C-type lectin domain family 18, member A	16	1	17
ENSG00000179088	C12orf42	Uncharacterized protein C12orf42	15	0	15
ENSG00000123329	ARHGAP9	Rho GTPase activating protein 9	14	0	14
ENSG00000144724	PTPRG	protein tyrosine phosphatase, receptor type, G	14	0	14
ENSG00000185630	PBX1	pre-B-cell leukemia homeobox 1	13	0	13
ENSG00000114850	SSR3	signal sequence receptor, gamma (translocon-associated protein gamma)	9	4	13
ENSG00000186153	WWOX	WW domain containing oxidoreductase	5	8	13
ENSG00000234906	APOC2	apolipoprotein C-II	12	0	12
ENSG00000180532	ZSCAN4	zinc finger and SCAN domain containing 4	12	0	12
ENSG00000144711	IQSEC1	IQ motif and Sec7 domain 1	12	0	12
ENSG00000138821	SLC39A8	solute carrier family 39 (zinc transporter), member 8	11	1	12
ENSG00000165995	CACNB2	calcium channel, voltage-dependent, beta 2 subunit	3	9	12
ENSG00000122859	NEUROG3	neurogenin 3	8	4	12
ENSG00000212128	TAS2R13	taste receptor, type 2, member 13	6	6	12
ENSG00000184999	SLC22A10	solute carrier family 22, member 10	11	0	11
ENSG00000183914	DNAH2	dynein, axonemal, heavy chain 2	11	0	11
ENSG00000125968	ID1	inhibitor of DNA binding 1, dominant negative helix-loop-helix protein	11	0	11
ENSG00000167962	ZNF598	zinc finger protein 598	10	1	11
ENSG00000181722	ZBTB20	zinc finger and BTB domain containing 20	3	8	11
ENSG00000091482	SMPX	small muscle protein, X-linked	10	0	10
ENSG00000117318	ID3	inhibitor of DNA binding 3, dominant negative helix-loop-helix protein	10	0	10
ENSG00000163694	RBM47	RNA binding motif protein 47	10	0	10
ENSG00000006007	GDE1	glycerophosphodiester phosphodiesterase 1	9	1	10
ENSG00000164104	HMGB2	high-mobility group box 2	8	2	10
ENSG00000250589	DUX4	double homeobox 4	2	8	10
ENSG00000130559	CAMSAP1	calmodulin regulated spectrin-associated protein 1	3	7	10
ENSG00000166501	PRKCB	protein kinase C, beta	4	6	10
ENSG00000186115	CYP4F2	cytochrome P450, family 4, subfamily F, polypeptide 2	4	6	10

Differentially expressed genes resulting from the general filtering of BMP7 data (905) were ranked according to the number of times a gene was up- and downregulated throughout the series of cell lines and time points. Only those genes with total rank value of at least 10 are listed.

## Discussion

In recent years, it has become increasingly accepted that the deregulation of mechanisms normally involved in developmental processes has tumorigenic effects in adult tissues. One example is the BMP family of growth factors, whose function in cancer physiology has been demonstrated in many tumor types, including breast cancer [9-11]. In spite of this, little is known about BMP target genes in the context of tumors. The transcriptional responses of breast cancer cells to BMP signaling have been studied only minimally. More precisely, the effects of BMP2 and BMP7 treatments on transcription in MCF-7 and MDA-MB-468 breast cancer cell lines, respectively, have been analyzed using cDNA microarrays of limited content (from several hundreds to 14, 500 gene probes) [28-30]. In this study, we have therefore set up an experimental procedure to identify potential BMP target genes in breast cancer by studying the effects of two BMP ligands, BMP4 and BMP7, on genome-wide gene expression. These two BMPs were selected based on their essential role in breast cancer, which we and others have demonstrated in recent years [9-11]. Both ligands are highly expressed in primary breast carcinomas as well as in breast cancer cell lines [12-14,16,17]. BMP7 expression was also shown to be associated with early bone metastasis [15]. Additionally, *in vitro *studies have implicated BMP4 and BMP7 as important regulators of proliferation and migration of breast cancer cells [14,18,20,21,31].

Our experimental approach allowed multiple types of analyses and revealed interesting insights into how the stimulation of BMP4 and BMP7 signaling influences the transcriptome of breast cancer cell lines. First of all, we showed relatively high numbers of DEPs as a result of BMP treatments, indicating a strong impact of BMP4 and BMP7 on the cell lines studied. Moreover, the transcriptional response to BMP4 was of a clearly higher magnitude than that induced by BMP7. Another aspect of the study was the opportunity to compare the effects of BMP signaling between cell lines. Interestingly, clear differences were seen in the amounts of DEPs, as well as in their expression patterns, as revealed by hierarchical clustering. BMP signaling pathways are regulated in a very complex manner and at many different levels, from the availability of BMP receptors, BMP ligands and BMP antagonists in the extracellular compartment to the presence or absence of various intracellular signal mediators and transcriptional co-activators or co-repressors [32,33]. Therefore, multiple factors influence the outcome of BMP signaling on the transcriptional level in a given cell. We have previously reported that all six BMP specific receptors (ACVR1, BMPR1A, BMPR1B, ACVR2A, ACVR2B, and BMPR2) are uniformly expressed among the breast cancer cell lines studied here [12]. Similarly, we have shown that SMAD4 is expressed and that phosphorylation of SMAD-1/5/8 is induced in these cell lines after BMP7 and BMP4 treatment [14,21]. Taken together, the expression profiles of BMP specific receptors or the mediators of the canonical intracellular pathway do not seem to have a major role in explaining the different transcriptional responses in the breast cancer cells. Nevertheless, due to the complexity of BMP signaling regulation, it is easy to understand that different cell lines may have different transcriptional responses to BMP stimulation. This observation highlights the importance of testing multiple cell lines when studying BMP signaling in cancer. An additional finding that could be inferred from our data is that induction of gene transcription, compared with inhibition, was the common response among those genes most frequently regulated by BMP4 and BMP7 in breast cancer cells. Likewise, previous microarray-based transcriptomic analyses of TGF-β and BMP have shown that induction of gene expression is the predominant response of mammalian cells to stimulation by these growth factors [28,30,33-35].

After BMP4 and BMP7 stimulation, the microarray analyses identified a large number of differentially expressed genes in our panel of cell lines. To explore the biological functions of these genes, GO enrichment analyses were performed. These revealed very similar results for both BMP ligands, namely, regulation of transcription and developmental processes. It seems, therefore, that the functions most prevalently influenced by BMP signaling in breast cancer cells do not differ remarkably from conventional roles that BMPs possess during development [36,37].

Synexpression groups are synchronously coexpressed gene sets, particularly apparent during embryonic development and in the response of cells to hormones and growth factors [38,39]. Our analyses unveiled that treatment of breast cancer cells with either BMP4 or BMP7 resulted in the coordinated expression of a group of genes (clusters A and B, respectively). Most interestingly, a considerable number of the genes in these two synexpression groups were common for the two ligands (group C). Moreover, our data indicated that treatment of a cell line with either BMP4 or BMP7 results in similar transcriptional responses of group C genes. We therefore hypothesize that group C represents molecular responses shared by the BMP4 and BMP7 signaling pathways. This finding prompted us to ask what functions these common genes fulfill in the cell. GO enrichment analysis of the genes in group C revealed that these genes are involved in two main biological processes, regulation of gene expression and regulation of development and morphogenesis. These results support the notion that genes known to regulate development also have functions that are important for the maintenance of cancer cells.

We also studied the temporal patterns of the transcriptional response after BMP treatment. The number of DEPs showed a tendency to increase with time, a trend previously noticed in transcriptome analysis of TGF-β family members in murine mammary epithelial cells and in breast cancer cells [29,35]. The DEPs could be grouped according to their temporal pattern of expression, varying from early to late responders. These temporal clusters were found in every cell line, and some of them even contained over a thousand gene elements. The next logical step was to explore whether there was a time-dependent shift in the distribution of gene functions. Although GO enrichment results were not obtained for all the probe clusters of all the cell lines, interesting features could be identified, especially in the case of the BMP4 data. Transcriptional regulation in the first 6 hours concentrated most notably on genes involved in developmental processes, metabolic processes, gene expression and signal transduction. Gene expression was also well-represented after 12 hours, while metabolism became by far the most prominent function at this time point. Most interestingly, 24 hours after BMP4 stimulation there was an evident overrepresentation of genes involved in cell proliferation, although this phenomenon was observed exclusively in MDA-MB-361 cells. All in all, the enriched biological functions indeed fluctuated in time and in a logical sequence, with regulation of gene expression and signal transduction leading to changes in metabolism and finally to regulation of cell proliferation, a phenotype relevant for cancer cell physiology. The fact that we did not see enrichment of cell proliferation-associated functions in more than one cell line could be due to differences in the speed of BMP signaling in different cell lines. Even though a longer experiment certainly could have clarified this issue, we concentrated our analysis on the first 24 hours after BMP treatment because we were interested primarily in the identification of BMP target genes.

As mentioned, one of the main goals of this study was to identify potential novel gene targets of BMP signaling relevant in breast cancer. We provided lists of candidate genes that are strongly and rather uniformly regulated by BMP4 or BMP7 throughout the cell lines and time points. Some of them, such as members of the Id family of inhibitors of DNA binding, are well-known BMP target genes [27,29]. Id proteins are transcription factors that regulate cell growth and differentiation [40], and all four members of the protein family play crucial roles in various aspects of normal and malignant breast biology [41]. Others are newly linked, in this work, to BMP signaling, and some of these genes have interesting connections with tumor biology, such as *PTPRG *or *DUSP2*. A positive feedback regulation where BMP treatment leads to increased expression of BMP antagonists is known to exist. In our study, no consistent expression changes were observed for any of the known BMP antagonists, such as noggin, gremlin, sclerostin and follistatin. Previous studies have shown a wide time window in the induction of e.g. noggin expression in different tissues after BMP treatment, ranging from 1 to 48 hours [42,43]. Thus it is possible that the feedback effect in the breast cancer cells was not evident at time points analyzed here.

Protein tyrosine phosphatases (PTPs) are key regulators of the cellular protein phosphorylation balance, critical in the control of a wide spectrum of physiological processes such as cell proliferation, differentiation, transformation, transport and locomotion. Subsequently, aberrations in phosphorylation processes play a major role in the pathogenesis of numerous diseases, including cancer [44,45]. PTPRG is a receptor-type PTP implicated as a candidate tumor suppressor gene in several types of tumors, including breast cancer [46,47]. In MCF-7 breast cancer cells, PTPRG inhibits proliferation and anchorage-independent growth and reduces tumor formation in a xenograft model [47,48]. Delayed cell cycle re-entry by increasing the level of cell cycle regulators p21 and p27 could explain the inhibitory effect of PTPRG on cell growth [47]. Based on the above, upregulation of PTPRG in BMP-stimulated cancer cells could contribute to the observed BMP-induced antiproliferative effect [14,21]. DUSP2 also belongs to the PTP family of phosphatases. It is a mitogen-activated protein kinase (MAPK) phosphatase (MKP) that dephosphorylates both threonine and tyrosine residues within target MAPKs leading to their deactivation. MAPK signaling controls cellular processes such as proliferation, differentiation, migration and apoptosis [48]. Therefore, abnormal MKP activity, and hence anomalous MAPK signaling, has important consequences for processes critical to the development and progression of human cancer. The role of DUSP2 in cancer has been examined in only a few studies, and data are controversial. Overexpression of DUSP2 expression was found in 37 of 39 malignant effusions from serous ovarian carcinoma patients and was associated with poor survival [49]. By contrast, decreased *DUSP2 *transcript levels were reported in cancerous breast, colon, lung, ovary, kidney and prostate tissues, and reduced DUSP2 protein levels were observed in cervical and colon cancer [50]. Additionally, DUSP2 suppression was associated with tumorigenesis and malignancy in colon cancer, and DUSP2 overexpression induced apoptosis and inhibited tumor growth in HeLa cells *in vitro *and in xenograft models [51].

## Conclusions

All in all, we show that BMP4 and BMP7 have strong effects on gene expression in breast cancer cells, and that this transcriptional response and its functional outcome follow a temporal sequence. Our data support the existence of a synexpression group of regulated genes that represent the core molecular responses shared by BMP4 and BMP7 signaling pathways. Additionally, we provide a list of potential novel BMP target genes relevant in breast cancer.

## Competing interests

The authors declare that they have no competing interests.

## Authors' contributions

ARM participated in the design of the study, carried out the interpretation of the data analyses and drafted the manuscript. ELA was responsible for conception and design of the project and participated in writing the manuscript. LS performed computational data analyses and participated in writing the manuscript. JK performed the microarray hybridizations. KN performed computational data analyses. SH supervised and coordinated the computational data analyses and participated in writing the manuscript. AK was responsible for conception and coordination of the project and participated in writing the manuscript. All authors read and approved the final manuscript.

## Pre-publication history

The pre-publication history for this paper can be accessed here:

http://www.biomedcentral.com/1755-8794/4/80/prepub

## Supplementary Material

Additional file 1 **List of probes in group C**. The expression values are represented as log2 ratios. Data was colored according to the accompanying color key. The data sheet contains 210 probes. Click here for file

Additional file 2 **GO enrichment results for group C genes**. Click here for file

Additional file 3 **Model-based clustering results and GO enrichment**. The probe clusters obtained are represented as well as the results of the GO enrichment analyses. GO terms are colored according to the accompanying color key. Click here for file

Additional file 4 **Most commonly regulated genes with rank 6 or higher**. The expression values are represented as log2 ratios. Data was colored according to the accompanying color key. The data sheets contain 608 and 281 genes for BMP4 and BMP7, respectively. Click here for file
